# Insulin and Exendin-4 Reduced Mutated Huntingtin Accumulation in Neuronal Cells

**DOI:** 10.3389/fphar.2020.00779

**Published:** 2020-05-28

**Authors:** Silvia Rea, David Della-Morte, Francesca Pacifici, Barbara Capuani, Donatella Pastore, Andrea Coppola, Roberto Arriga, Aikaterini Andreadi, Giulia Donadel, Nicola Di Daniele, Alfonso Bellia, Davide Lauro

**Affiliations:** ^1^Department of Systems Medicine, University of Rome Tor Vergata, Rome, Italy; ^2^Department of Human Sciences and Quality of Life Promotion, San Raffaele Roma Open University, Rome, Italy; ^3^Department of Neurology, Miller School of Medicine, The Evelyn F. McKnight Brain Institute, University of Miami, Miami, FL, United States; ^4^Department of Clinical Sciences and Translational Medicine, University of Rome Tor Vergata, Rome, Italy; ^5^Department of Medical Sciences, Fondazione Policlinico Tor Vergata, Rome, Italy

**Keywords:** Huntington disease, neuronal survival, insulin, exendin-4, mutated huntingtin

## Abstract

Patients with diabetes mellitus (DM) are more prone to develop cognitive decline and neurodegenerative diseases. A pathological association between an autosomal dominant neurological disorder caused by brain accumulation in mutated huntingtin (mHTT), known as Huntington disease (HD), and DM, has been reported. By using a diabetic mouse model, we previously suggested a central role of the metabolic pathways of HTT, further suggesting the relevance of this protein in the pathology of DM. Furthermore, it has also been reported that intranasal insulin (Ins) administration improved cognitive function in patients with neurodegenerative disorders such as Alzheimer disease, and that exendin-4 (Ex-4) enhanced lifespan and ameliorated glucose homeostasis in a mouse model of HD. Although antioxidant properties have been proposed, the underlying molecular mechanisms are still missing. Therefore, the aim of the present study was to investigate the intracellular pathways leading to neuroprotective effect of Ins and Ex-4 hypoglycemic drugs by using an *in vitro* model of HD, developed by differentiated dopaminergic neurons treated with the pro-oxidant neurotoxic compound 6-hydroxydopamine (6-ohda). Our results showed that 6-ohda increased mHTT expression and reduced HTT phosphorylation at Ser421, a post-translational modification, which protects against mHTT accumulation. Pre-treatment with Ins or Ex-4 reverted the harmful effect induced by 6-ohda by activating AKT1 and SGK1 kinases, and by reducing the phosphatase PP2B. AKT1 and SGK1 are crucial nodes on the Ins activation pathway and powerful antioxidants, while PP2B dephosphorylates HTT contributing to mHTT neurotoxic effect. In conclusion, present results highlight that Ins and Ex-4 may counteract the neurotoxic effect induced by mHTT, opening novel pharmacological therapeutic strategies against neurodegenerative disorders, with the main focus on HD, still considered an orphan illness.

## Introduction

A significant pathological association between type 2 diabetes mellitus (T2DM) and progression on the decline in cognitive function and neurodegenerative diseases has been reported (Bangen et al., 2015). Longitudinal prospective studies conducted in patients with Alzheimer disease (AD) (Arnold et al., 2018), Parkinson disease (Pagano et al., 2018), and Huntington disease (HD) support this evidence (Montojo et al., 2017), suggesting that common molecular pathways may be involved in the mutual development of T2DM and the aforementioned illnesses.

By using a proteomic approach in a diabetic mouse model, we demonstrated a novel central role of huntingtin protein (HTT) in metabolism and in glucose homeostasis (Capuani et al., 2015). An expansion of the CAG (polyQ) repeats in the gene encoded for HTT caused HD, which is an orphan autosomal dominant disease leading to neuronal death (apoptosis), dementia, and movement disorders (Jimenez-Sanchez et al., 2017). However, so far, molecular mechanisms linking T2DM and HD need to be fully elucidated (Montojo et al., 2017). The increase in cellular oxidative stress, characteristic of both diseases, has been indicated among the main processes (Montojo et al., 2017). Moreover, a positive associations between HTT and kinases activated by insulin (Ins), such as AKT1 and serum‐ and glucocorticoid‐induced kinase-1 (SGK1), and their downstream pathways, have also been suggested (Rangone et al., 2004). AKT1 and SGK1 are powerful antioxidants (Ferrelli et al., 2015) and were already implicated in HD pathogenesis (Bowles and Jones, 2014). By further investigating involvement of these kinases on HD, this will allow us to open novel therapeutic strategies against this orphan disease. Drugs that counteract T2DM may be able to cure and delay the progression of HD. Insulin and exendin-4 (Ex-4) have already been proven to exert neuroprotection in experimental models of AD and HD (Rangone et al., 2004) (Martin et al., 2009; Zhang et al., 2015). Therefore, in the present study, we sought to investigate the mechanisms underlying the protective role of both Ins and Ex-4 against mutated HTT-inducing toxicity by an *in vitro* model of HD.

## Materials and Methods

### Cell Culture and Differentiation

Human neuroblastoma cell line SH-SY5Y were purchased from ATCC (American Type Culture Collection Manassas, VA, USA). Cells were cultured in Dulbecco's Modified Eagle Medium/Nutrient Mixture F-12 medium (DMEM F-12), supplemented with 10% heat-inactivated fetal bovine serum (FBS, Corning), 2 mM of glutamine, and 100 U/ml of penicillin/streptomycin (Thermo Fisher Scientific®, Waltham, MA, USA). Cells were maintained at 37°C in humidified air containing 5% CO_2_.

Cell differentiation was performed according to Lopes et al., (Lopes et al., 2017). Briefly, 4x10^5^ cells/well were seeded in a six well plate, using 10% FBS medium. After 24 h (designed as day 1), medium was removed and replaced with 1% FBS medium supplemented with 10 μM of all-trans-retinoic acid (RA, Sigma Aldrich). Medium was replaced every 2 d for 6 d when the presence of neuronal differentiation markers were verified and cells were used for experiments. Morphological changes, due to differentiation, were monitored by using an inverted microscope at 100X and 40X of magnification.

### Treatments

Cell neurotoxicity was induced by using 6-hydroxydopamine (6-ohda, Sigma Aldrich), as previously reported (Lopes et al., 2017). Briefly, cells were seeded in a 96 multi-well plate (2x10^4^ cells/well) and, following the differentiation process as previously reported, were treated with increasing concentration of 6-ohda (10–30–50–75–100 µM), for 24 h. In order to avoid 6-ohda oxidation, as reported by manufacturer's protocol, we dissolved the powder by adding the antioxidant sodium metabisulfite at 0.1%. Once we assessed the neurotoxic effects, by using 6-ohda (30 µM) for 24 h, SH-SY5Y cells were seeded in 6 multiwell plate (4x10^5^ cells/well, for western blot and FACS analysis) or in a 96 multi-well plate (2x10^4^ cells/well, for cell toxicity assay), and differentiated. Subsequently, cells were pre-treated with Ex-4 (Sigma Aldrich) (300 nM) (Eakin et al., 2013) for 2 h, or with Ins (Sigma Aldrich) (100 nM) (Ramalingam and Kim, 2017) for 1 h, and then 6-ohda was administered.

### Cell Viability Assay MTT

Cell viability was evaluated through MTT (3-(4,5-dimethylthiazol-2-yl)-2,5-diphenyltetrazolium bromide) colorimetric assay (Sigma Aldrich), following manufacturer's protocol. Briefly, SH-SY5Y were seeded and treated as described in the previous section. Then cells were incubated at 37°C, with medium containing MTT 5 mg/ml; after 3 h, DMSO (dimethyl sulfoxide) (Sigma Aldrich) was added in the medium and MTT-formazan conversion was evaluated by measuring sample absorbance at 570 nm.

### Gene Expression

Total RNA was isolated from SH-SY5Y by using Trizol reagent (Thermo Scientific) as previously reported by Pacifici et al (Pacifici et al., 2014). Briefly, two and one-half micrograms of total RNA was reverse transcribed into cDNA using a High Capacity cDNA Archive Kit (Applied Biosystems). Qualitative qRT-PCR was performed using an ABI PRISM 7500 System and TaqMan reagents (Applied Biosystems). Each reaction was performed in duplicate using standard conditions, and results were normalized with glyceraldehyde 3-phosphate dehydrogenase (GAPDH). The relative expression of VMAT1 (vesicular monoamine transporter 1) was calculated using the comparative ΔΔCT method, and the values were expressed as 2^−ΔΔCT^ (Livak and Schmittgen, 2001). VMAT1 and GAPDH sequence primers are inventoried and under patent protection.

### Apoptosis Cell Death Analysis

SH-SY5Y were seeded and treated as previously described. Subsequently, cells were collected, centrifuged at 1,600 rpm for 5 min and fixed in a 70% ethanol solution for 45 min at +4°C. Then, were centrifuged at 1,600 rpm for 5 min and resuspended in a 25 µg/ml propidium solution, containing 0.05% sodium citrate and 0.1% of TRITON X-100. Apoptotic cells in sub G1 phase of the cell cycle (hypodiploid nuclei) were identified and quantified by flow cytometry as reported by Riccardi et al, (Riccardi and Nicoletti, 2006).

### Western Blot Analysis

Differentiated SH-SY5Y were lysed in cold lysis buffer containing 20 mM Tris (pH 7,6), 137 mM NaCl, 1,5% Nonidet P40, 1 mM MgCl_2_, 1 mM CaCl_2_, 10% glycerol, 2 mM PMSF (phenylmethanesulfonyl fluoride), phosphatases, and proteases inhibitors cocktail 1X (Sigma Aldrich). Samples were maintained on ice for 30 min and then centrifuged at 14,000 rpm for 30 min. Supernatants, containing proteins extracts were collected and protein concentration were determined by colorimetric Bradford assay (Bio-Rad Laboratories), using BioPhotometer™ Plus instrument (Eppendorf). Protein samples were used for Western blot analysis or stored at −80° C. Then, 50 µg of protein lysates were loaded on pre-cast 4–12% or 3–8% gels (Thermo Scientific) separated by SDS-PAGE and transferred to nitrocellulose membranes using Trans Blot TurboTM Transfer System (Bio-Rad Laboratories). Subsequently, membranes were probed with the following primary antibodies: mouse anti vinculin, mouse anti PP2B (1:200, Santa Cruz Biotechnology), mouse anti actin, rabbit anti PARP1, rabbit anti TH, rabbit anti HTT D7F7, rabbit anti AKT1 phospho-Tyr 473, rabbit anti AKT1 (1:1,000, Cell Signaling), mouse anti HTT mab2166, rabbit anti SGK1 (1:500 and 1:1,000 respectively, Millipore), and rabbit anti HTT phospho-Ser 421 (1:500, Abcam). The secondary antibodies were purchased from Jackson Immunoresearch and used at a dilution of 1:10000. The antigen-antibody complexes were next detected with enhanced chemiluminescence (ECL) reagent (GE Healthcare) followed by exposure of the ChemiDoc Touch (Bio-Rad Laboratories). A densitometric analysis was performed by using Image LabTM Software (Bio-Rad Laboratories).

### Statistical Analysis

Data were analyzed by using GraphPad Prism 5 (La Jolla, CA, USA). All data were expressed as mean ± SEM, as indicated. Statistical analysis was performed by unpaired one-tailed Student's t-test. Values of p<0.05 were considered statistically significant.

## Results

### Generation of an In Vitro Model of HD

To generate an *in vitro* model of HD, human neuroblastoma SH-SY5Y cells were used. SH-SY5Y cells were already employed to mimic a cellular model of HD based on the *in vitro* accumulation of HTT (Min et al., 2013).

**Figure 1:**
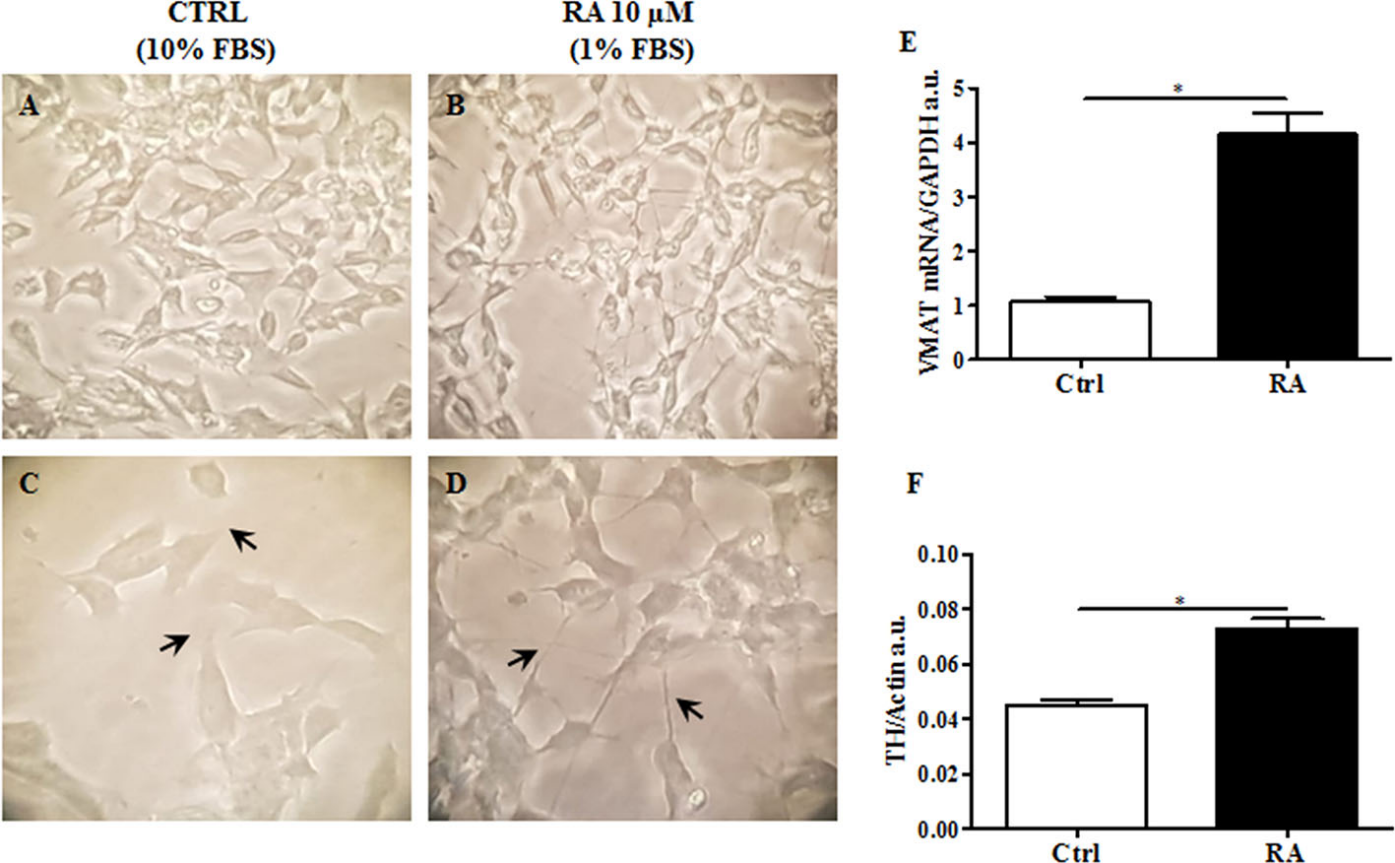
RA (retinoic acid) induced SH-SY5Y differentiation. Non differentiated SH-SY5Y, were cultured in 10% FBS enriched medium analyzed after 6 d at 40X **(A)** and 100X of magnification **(C)**. SH-SY5Y were differentiated by using RA 10 μM, for 6 d in 1% FBS enriched medium, and analyzed 40X **(B)** and 100X of magnification **(D)**. Differentiated cells showed extended neurites, forming a neuronal interconnection network (indicated by arrows). **(E)** Expression analysis of vesicular monoamine transporter 1 (VMAT1), a neuronal differentiation marker, was carried out by using glyceraldehyde 3-phosphate dehydrogenase (GAPDH) as internal control. VMAT1 was significantly increased in RA treated cells. **(F)**. Protein expression levels of tyrosine hydroxylase (TH) were evaluated. Actin was used as loading control. TH protein expression was significantly enhanced in RA treated cells. Graphs illustrate three separate studies, all yielding similar results (n=3). Data are reported as mean±SEM. *p < 0.05.

Since this cell line displays an immature fibroblast-like phenotype not suitable for a translational study (Santillo et al., 2014), we induced differentiation into neuronal-like phenotype. Based on Lopes and colleagues (Lopes et al., 2017), retinoic acid (RA), was used to differentiate SH-SY5Y (Lopes et al., 2017). Cells were treated with RA 10 μM for 6 d, as previously described in ***Material and Methods***. Then, while non-treated cells maintained unpolarized cell bodies and few short neurites (**Figures 1A, C**), RA treated cells showed more pyramidal cell bodies and extensive and developed neurites, typical of neuronal cells (**Figures 1B, D**).

In order to validate the differentiation process, vesicular monoamine transporter 1 (VMAT1/SLC18A1), a marker for synaptic function (Wimalasena, 2011; Korecka et al., 2013), and tyrosine hydroxylase (TH), a specific dopaminergic neuronal marker (Daubner et al., 2011), were both evaluated. As presented in **Figure 1E**, VMAT1/SLC18A1 gene expression significantly increased in cells following RA administration, compared to control cells (p<0.05). VMAT1/SLC18A1 is also a monoamine transporter, which includes dopamine. Since dopaminergic neurons are those most affected by neurodegeneration in HD (Walker, 2007), we believe that the present cellular model including these neurons may increase the impact of present results. Accordingly, TH expression levels significantly enhanced in RA treated cells as compared to control cells (p<0.05) (**Figure 1F**). These results suggest that RA, by conferring a specific dopaminergic neuronal phenotype to SH-SY5Y cells may be used to generate a cell line to study HD.

**Figure 2:**
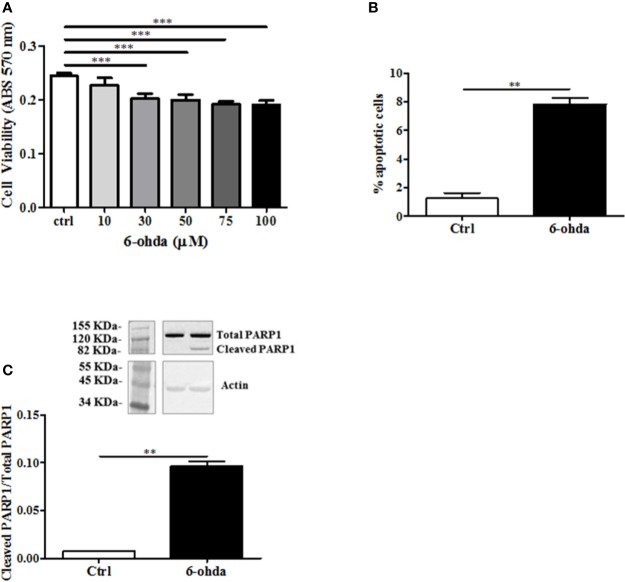
6-Hydroxydopamine (6-ohda) administration reduced cell viability and induced apoptotic death. **(A)** Cell viability was measured by MTT colorimetric assay after 24 h of treatment with 6-ohda, at indicated concentrations. 6-ohda significantly reduced cell viability in SH-SY5Y in a dose dependent manner. **(B)** Apoptosis was evaluated 24 h after 30 µM of 6-ohda administration. Cells were stained with propidium iodide (PI) and analyzed: 6-ohda administration induced a significant increase in cell apoptosis. **(C)** Poly [ADP-ribose] polymerase 1 (PARP1) cleavage, was increased in SH-SY5Y treated with 30 µM of 6-ohda for 24 h. Total PARP1 was used as loading control. Graphs illustrate three separate studies, all yielding similar results (n=3). Data are reported as mean±SEM. **p < 0.005, ***p < 0.001. MTT: (3-(4,5-dimethylthiazol-2-yl)-2,5-diphenyltetrazolium bromide) tetrazolium.

6-Hydroxydopamine is a specific neurotoxic molecule able to induce death in dopaminergic neurons (Breese et al., 2005). In postmortem studies, brains from HD patients have shown to loose predominantly dopaminergic neurons as compared to control subjects (Rangel-Barajas and Rebec, 2016). Then, we hypothesized a role of 6-ohda in mimicking this neuronal damage observed in these patients. The toxicity of 6-ohda in neurons and the related accumulation of mutated huntingtin (mHTT) depend on the cellular increase in oxidative stress levels (Sulzer and Zecca, 2000). Therefore, differentiated cells were treatedwith increasing concentrations (from 10 to 100 μM) of 6-ohda for 24 h. Thereafter, cell viability was assessed using MTT colorimetric assay. Results reported in **Figure 2A** presented as 30 μM of 6-ohda, which was the lowest concentration to induce a significant reduction in cell viability after 24 h of treatment (p<0.001). This concentration was selected for the subsequent experimental procedures.

The measurement in cell viability does not explain the whole mechanism underlying the cellular death that instead may be multiple and variable (Pattison et al., 2006). We then tested apoptotic cell death in the same experimental conditions. As shown in **Figure 2B**, 6-ohda administration induced apoptosis in differentiated SH-SY5Y compared to non-treated differentiated control cells (p<0.005). Moreover, to further validate pro-apoptotic effect of 6-ohda, we measured PARP1 cleavage, a key step in apoptotic process (Chaitanya et al., 2010). Differentiated cells treated with 6-ohda showed a significant increase in PARP1 cleavage and activation (**Figure 2C**), confirmed induction of apoptotic the process.

**Figure 3:**
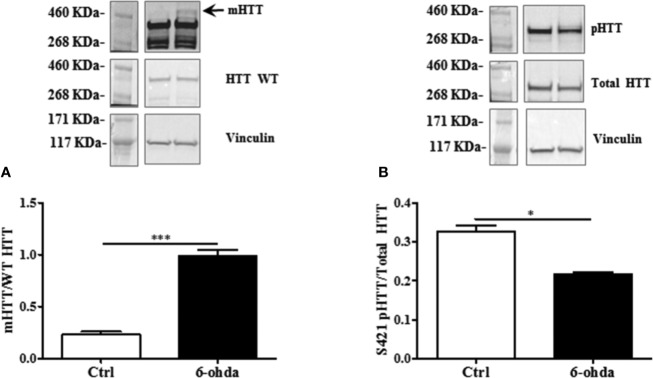
6-Hydroxydopamine (6-ohda) administration increased mutated huntingtin (mHTT) by reducing HTT phosphorylation at Ser421. **(A)** Protein expression levels of mHTT was significantly increased following 6-ohda administration. **(B)** phosphorylated huntingtin (pHTT) [at Ser421 (S421)] levels were reduced by the treatment with 6-ohda. HTT WT (huntingtin wild type) and total HTT were respectively used as loading controls. Graphs illustrate three separate studies, all yielding similar results (n=3). Data are reported as mean±SEM. *p < 0.05, ***p < 0.001.

To evaluate whether 6-ohda administration mimicked HD pathogenesis, we measured mHTT protein accumulation. Following 6-ohda stimulation, mHTT expression was evaluated by western blot analysis. As reported in **Figure 3A**, 6-ohda significantly enhances mHTT levels (p<0.001), which is the most relevant pathogenic mechanism leading to HD (Jimenez-Sanchez et al., 2017). Phosphorylation of HTT in Serine421 (S421-pHTT) conversely plays a pivotal role in neuroprotective mechanisms against HD by blunting mHTT accumulation/neurotoxicity (Warby et al., 2009; Kratter et al., 2016). Accordingly, in our experimental model S421-pHTT levels were significantly reduced following 6-ohda treatment as compared to control cells (p<0.05) (**Figure 3B**). These data suggest that 6-ohda, by inducing either mHTT increase than S421-pHTT decrease, may be considered a useful neurotoxic stimulus to study *in vitro* molecular mechanisms responsible for HD.

### Neuroprotective Effect of Ex-4 and Ins Against 6-ohda-Induced Apoptosis

**Figure 4:**
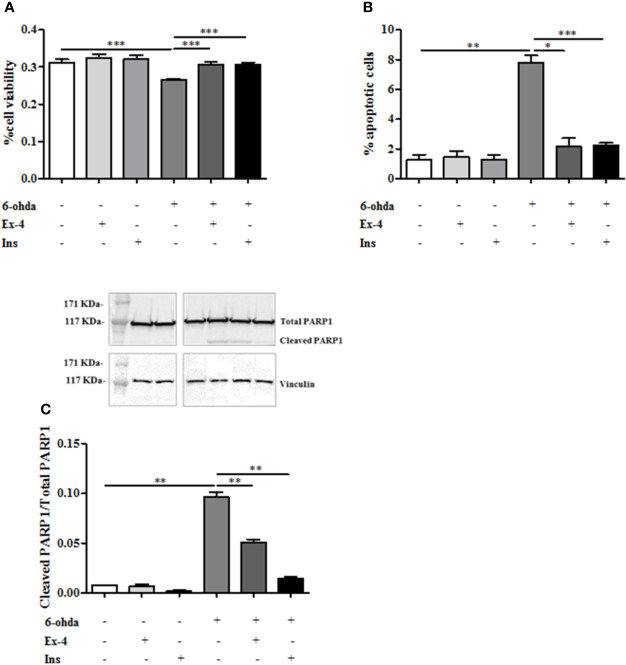
Exendin-4 (Ex-4) or insulin (Ins) pre-treatment restore cell viability and blunted apoptosis. **(A)** Cell were treated with 300 nM of Ex-4 for 2 h, or with 100 nM of Ins for 1 h before neurotoxic insult with 6-hydroxydopamine (6-ohda) (30 μM, 24 h). Cell viability, analyzed by MTT assay, revealed a significant increase of viability in both Ex-4 and Ins pre-treated cells relative to cells treated with 6-ohda alone. **(B)** In the same experimental conditions of Panel A, apoptosis was evaluated by staining cells with propidium iodide (PI). Both in Ex-4 and Ins pre-treated cells a reduction in apoptosis was observed, compared to cells treated with 6-ohda alone. **(C)** Poly [ADP-ribose] polymerase 1 (PARP1) cleavage, was reduced following Ex-4 or Ins administration. Total PARP1 was used as loading control. Graphs illustrate three separate studies, all yielding similar results (n=3). Data are reported as mean±SEM. *p < 0.05, **p < 0.005, ***p < 0.001.

Ex-4 is an agonist of glucagon-like peptide-1 (GLP-1) receptor which acts by promoting Ins secretion (Furman, 2012). In order to test whether Ex-4 and Ins exert a protective effect against neurotoxicity induced by 6-ohda, differentiated cells were pre-treated with Ex-4 (300 nM, for 2 h) (Eakin et al., 2013), or Ins (100 nM, for 1 h) (Ramalingam and Kim, 2017) before 6-ohda (30 μM) administration for 24 h. Afterward, cell viability was evaluated by MTT colorimetric assay. **Figure 4A** presented with both Ex-4 and Ins significantly improved cell viability, which was impaired by 6-ohda, as compared to control cells (p<0.001). As expected, both Ex-4 and Ins decreased apoptotic cell death induced by 6-ohda (p<0.05 and p<0.001, respectively) (**Figure 4B**). This neuroprotective effect was confirmed by the reduction on PARP1 cleavage (p<0.005) (**Figure 4C**) induced by both Ex-4 and Ins, supporting the pro-survival effect of these compounds.

### Ex-4 and Ins Prevented Increase of Mutated HTT by Promoting HTT Phosphorylation Via AKT1, SGK1, and PP2B Modulation

**Figure 5:**
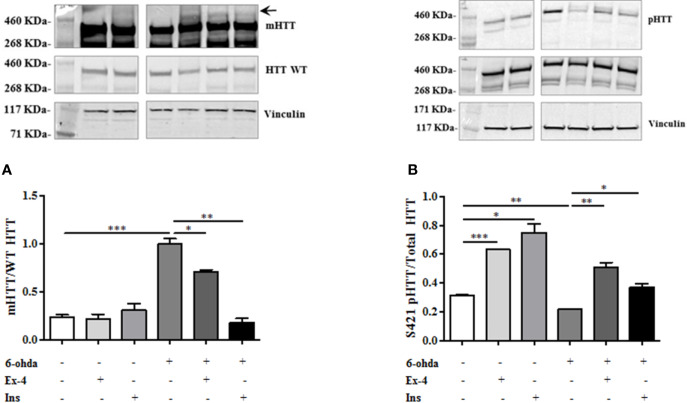
Exendin-4 (Ex-4) or insulin (Ins) pre-treatment blunt mutated huntingtin (mHTT) and enhanced pHTT levels. **(A)** Ex-4 300 nM for 2 h, or Ins 100 nM for 1 h pre-treatment before neurotoxic insult with 6-hydroxydopamine (6-ohda) (30 μM, 24 h), significantly blunted mHTT induced by 6-ohda. **(B)** In the same experimental conditions of panel A, both Ex-4 and Ins increased pHTT protein expression levels compared to cells treated with 6-ohda alone. HTT WT and total HTT were respectively used as loading controls. Graphs illustrate three separate studies, all yielding similar results (n=3). Data are reported as mean±SEM. *p < 0.05, **p < 0.005, ***p < 0.001.

We also tested whether Ex-4 and Ins exerted positive effect on mHTT accumulation levels. Thus, differentiated SH-SY5Y were pre-treated with Ex-4 or Ins, as previously described, and then treated with 6-ohda for 24 h. As shown in **Figure 5A**, pre-treatment with Ex-4 or Ins significantly blunted the increase in mHTT levels induced by 6-ohda (p<0.05 and p<0.005, respectively) in dopaminergic neurons. As previously demonstrated, 6-ohda reduced S421-pHTT levels that in neurons protects against accumulation of poly(Q)-mHTT (Metzler et al., 2010).

As expected, both Ex-4 and Ins significantly restored S421-pHTT levels as compared to those observed after 6-ohda administration (p<0.005 and p<0.05, respectively) (**Figure 5B**), suggesting that both drugs induced neuroprotection through HTT phosphorylation in S421.

**Figure 6:**
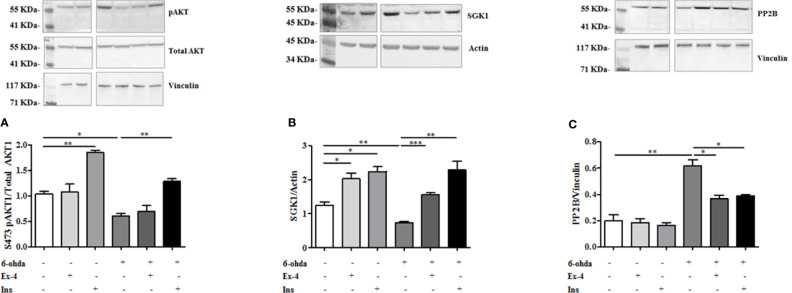
Protein expression analysis of pAKT1, SGK1, and PP2B. **(A)** Cell were treated with 300 nM of Ex-4 for 2 h, or with 100 nM of Ins for 1 h before neurotoxic insult with 6-hydroxydopamine (6-ohda) (30 μM, 24 h). pAKT1 levels were significantly increased following Ins (but non Ex-4) administration compared to cells treated with 6-ohda alone. Total AKT1 was used as loading control. **(B)** In the same experimental conditions of panel A, SGK1 protein expression were enhanced following Ex-4 or Ins supplementation. Vinculin was used as loading control. **(C)** A significant increase of PP2B occurred in 6-ohda treated cells, while in Ex-4 or Ins pre-treated cells, a significant reduction was observed. Vinculin was used as loading control. Graphs illustrate three separate studies, all yielding similar results (n=3). Data are reported as mean±SEM. *p < 0.05, **p < 0.005, ***p < 0.001.

The role of AKT1 and SGK1 in promoting HTT phosphorylation in S421 is known (Humbert et al., 2002; Rangone et al., 2004). As reported in **Figure 6A**, AKT1 phosphorylation at S473, and subsequent AKT1 activation, decreased after 6-ohda administration, as compared to control non-treated cells (p<0.05). Pre-treatment with Ins enhanced AKT1 levels both in basal condition (p<0.005), and after noxious stimulus (p<0.005), further remarking Ins specificity for this kinase. No significant results were present for Ex-4. These data suggest that Ins, in an AKT1-dependent manner, may exert a neuroprotective effect by promoting HTT phosphorylation.

Similarly to AKT1 also SGK1 protein expression was reduced compared to control cells following 6-ohda administration (p<0.005) (**Figure 6B**). Instead, conversely to AKT1, either Ex-4 than Ins significantly increased expression of SGK1, either in basal conditions (p<0.05) than after stimulation with 6-ohda (p<0.005) (**Figure 6B**). Our data suggest a significant involvement of AKT1 and SGK1 kinases in the Ex-4 and Ins induced neuroprotection mediated by S421-pHTT.

However, HTT phosphorylation in S421 is not only regulated by AKT1 and SGK1 kinases, but also by PP2B phosphatase (Saudou and Humbert, 2016). As shown in **Figure 6C** a substantial modulation of phosphatase levels was evident. In particular, PP2B expression significantly increased in cells treated with 6-ohda, suggesting that reduced levels of S421-pHTT induced by 6-ohda could be also associated to an increase in its dephosphorylation by PP2B. It is also worth noting as pre-treatment with Ex-4 or Ins prevented the increase in phosphatase induced by 6-ohda (**Figure 6C**), demonstrating as the neuroprotective effect associated to these hypoglycemic agents can be also mediated by their ability in reduce PP2B expression.

## Discussion

**Figure 7:**
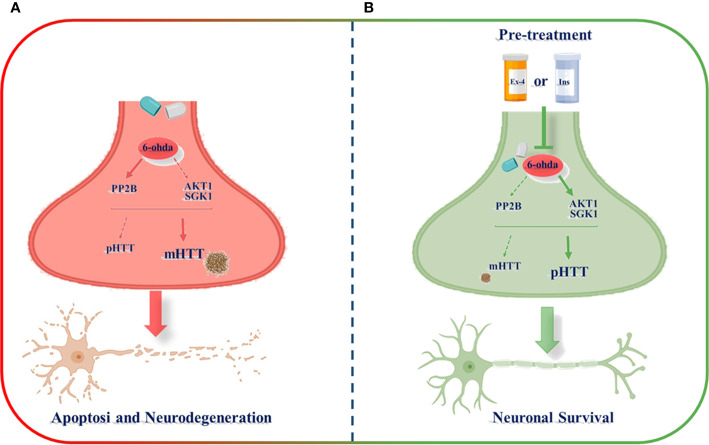
Schematic representation of exendin-4 (Ex-4) and insulin (Ins) neuroprotective effect against the neurotoxicity induced by 6-hydroxydopamine (6-ohda). **(A)** 6-ohda neurotoxic effect, characterized by enhanced mutated huntingtin (mHTT) levels in spite of pHTT, was mediated by the reduced activation of both AKT1 and SGK1 kinases, and by the increase in PP2B levels. Conversely, the pre-treatment with Ex-4 or Ins **(B)** restore neuronal survival by counteracting mHTT and PP2B toxicity and by promoting pHTT levels. BioRender software has been used.

In the present study, by the development of an experimental *in vitro* model commonly used to investigate Parkinson's disease (Chen et al., 2020), we generated a novel *in vitro* model consisting of differentiated dopaminergic neurons in order to explore possible molecular mechanisms underlying physiopathology of HD. Through this model, we found as differentiated human neuroblastoma cell line treated with a neurotoxic stimulus, 6-ohda, increased levels of cellular apoptosis, and mHTT protein accumulation that are associated, in turn, to low expression of HTT phosphorylation. Pre-treatment with two known hypoglycemic drugs, Ex-4 and Ins, resulted in neuronal protection by reducing mHTT levels and by restoring HTT phosphorylation, mainly through modulation of specific kinases, like AKT1, SGK1, and PP2B (**Figure 7**).

A pathological link between DM and HD has been already reported in literature (Montojo et al., 2017). Although HD is a “typical” neurodegenerative disorder characterized by a trinucleotide expansion (CAG: encoding glutamine ≥36 repeats) located in exon 1 of the *HTT* gene, which causes brain and peripheral defects, its impact in the endocrine system and in glucose metabolism is evident (Montojo et al., 2017). However, whether the dysfunction in the pathways regulating glycemic homeostasis triggers brain proteinaceous accumulation or vice versa is still not clear. What is known so far is that HTT is involved in both HD and T2DM (Capuani et al., 2015) and that this implication is further validated by present results.

As also suggested by the present results, the mechanism linked to mHTT neurodegeneration might be modulated by specific kinases, which when activated regulate metabolism and oxidative stress. In dopaminergic neurons, AKT1 levels were significantly blunted by toxic stimulus and restored after treatment with Ins. A study conducted on lymphoblasts and lymphocytes from HD patients, detected modifications of AKT1 expression and activity, confirming a dysregulation of this kinase in HD (Colin et al., 2005). Interestingly, AKT1 was cleaved into an inactive form by caspase-3, suggesting a pro-apoptotic mechanism underlying HD development, in agreement with our findings (Colin et al., 2005). An *in vitro* study conducted in nigrostriatal neurons showed that Ins like growth factor 1 (IGF-1) inhibited mHTT-induced cell death through AKT1 activation induced by S421-phosphorylation (Humbert et al., 2002), further supporting our evidences. Moreover, this mechanism abrogated polyQ-HTT pro-apoptotic activity (Humbert et al., 2002).

SGK1 is highly homologous with AKT1 and may be activated as alternative savage pathway by Ins when AKT1 is impaired (Lauro et al., 2015). Here, we demonstrated that this kinase is triggered either by Ex-4 or Ins and, when activated, reduces levels of mHTT. In previous *in vitro* studies, by inducing cellular transgenic activation of SGK1, we demonstrated as this kinase protected kidney cells from apoptosis (Pastore et al., 2015), and endothelial cells from oxidative stress and hyperglycemia (Ferrelli et al., 2015). Interestingly, in striatal neurons, according to present findings, SGK1 phosphorylates HTT at S421 leading to a reduction in mHTT accumulation and toxicity (Rangone et al., 2004).

Increase in AKT1 and SGK1 levels following Ex-4 and Ins treatment are also associated to a corresponding reduction in PP2B levels. The suggested common pathway among these three kinases (Wen et al., 2003; Schweitzer et al., 2012), along with the effect of AKT1, SGK1, and PP2B on HTT (Bowles and Jones, 2014; Saudou and Humbert, 2016), suggest that the one investigated in the present study may be a candidate pathway on the pathogenetic crosslink between DM and HD. However, further studies to validate our hypothesis are imperative, especially by using experimental models which include GABAergic neurons that, when destroyed, lead to over-activity of nigrostriatal dopaminergic neurons causing a down-regulation of dopamine receptors in striatum, a clinical hallmark of HD (Rosas-Arellano et al., 2018).

HD mice treated with Ex-4 ameliorated abnormalities in peripheral glucose regulation by suppressing cellular pathology in both brain and pancreas (Martin et al., 2009). Moreover, Ex-4 also improved pancreatic morphology, motor coordination, and increased lifespan in mice (Martin et al., 2012). Similarly, treatment with Ins in HD mice improved mitochondrial function and reduced mitochondrial oxidative stress induced by mHTT accumulation (Ribeiro et al., 2014). Furthermore, a Clinical Trial known as SNIFF (ID: NCT01767909) suggest that Ins administered intranasal improved cerebral Ins sensitivity allowing a decrease in proteinaceous brain formation. Together, the evidence strongly support that specific hypoglycemic drugs may be useful for prevention and cure of HD.

However, we need to acknowledge limitations of the present study. We did not assess any link between glucose homeostasis and HD, but we focused more on the cellular and molecular mechanisms. Since this is an *in vitro* study, unfortunately, it was not possible to assess physiological variables that may interact with the effects of Ins and Ex-4. The major strengths of the present study are the novelty of the model including differentiated dopaminergic neurons, to study *in vitro* HD, and the analysis of the kinases involved in the possible link between glucose homeostasis and HD.

In conclusion, present results suggest a novel potential role of cellular kinases that when activated by Ex-4 and Ins may induce neuroprotection, therefore opening a unique therapeutic strategy against HD, still considered an orphan illness.

## Data Availability Statement

The raw data supporting the conclusions of this article will be made available by the authors, without undue reservation, to any qualified researcher.

## Author Contributions

SR and DD-M contributed conception and design of the study. SR, BC, and FP performed experimental procedures. AC, DP, RA, and AA contributed data analysis and interpretation. GD, ND, and AB performed the statistical analysis. SR and FP wrote the first draft of the manuscript. DD-M, FP, and DL wrote sections of the manuscript. All authors contributed to manuscript revision, read and approved the submitted version.

## Funding

This work has been supported by following grants: Fondazione Roma NCDS-2013-00000331 - Sarcopenia and Insulin Resistance in the Elderly; Age-Associated Inflammation as a Shared Pathogenic Mechanism and Potential Therapeutic Target; Fondazione Roma - Diabetes Mellitus, Regenerative and Reparative Processes, and Improvement of Pancreatic Beta Cell Function: Role of Bone Marrow-Mesenchymal Stem Cells, MicroRNAs, M2 Macrophages and Myeloid Derived Suppressor Cells; Fondazione Umberto Di Mario; ASI N 2013-084-R0, COREA Research Project, Italian Space Agency; The Evelyn F. McKnight Brain Institute; PRIN 2015 # 2015373Z39_009 “Pancreatic β-cell identity, glucose sensing and the control of insulin secretion”, PRIN 2017, #201793XZ5A_004 “Metabolic therapy of immuno-inflammation: in search for the best strategy to counteract type 2 diabetes and its complications”.

## Conflict of Interest

The authors declare that the research was conducted in the absence of any commercial or financial relationships that could be construed as a potential conflict of interest.

## Abbreviations

T2DM, Type 2 diabetes mellitus; AD, Alzheimer disease; HD, Huntington disease; HTT, huntingtin; mHTT, mutated huntingtin; pHTT, phosphorylated huntingtin; SGK1, Serum‐ and glucocorticoid‐induced kinase-1; PP2B, Serine/threonine-protein phosphatase 2B; 6-ohda, 6-hydroxydopamine; Ex-4, Exendin-4; Ins, Insulin; RA, Retinoic acid; VMAT1/SLC18A1, Vesicular monoamine transporter 1; TH, Tyrosine hydroxylase.
